# Reliability and Feasibility of the Self-Administered ISTH-Bleeding Assessment Tool

**DOI:** 10.1055/s-0039-3400483

**Published:** 2019-11-29

**Authors:** Marieke C. Punt, Maaike W. Blaauwgeers, Merel A. Timmer, Paco M.J. Welsing, Roger E.G. Schutgens, Karin P.M. van Galen

**Affiliations:** 1 Van Creveldkliniek, University Medical Center Utrecht, University Utrecht, Utrecht, The Netherlands; 2 Department of Rheumatology and Clinical Immunology, University Medical Center Utrecht, University Utrecht, Utrecht, The Netherlands

**Keywords:** self-BAT, ISTH-BAT, bleeding tendency, congenital platelet defects

## Abstract

**Introduction** Standardized bleeding assessment tools (BATs), such as the International Society for Thrombosis and Hemostasis (ISTH)-BAT, are screening instruments used during the diagnostic workup of suspected bleeding disorders. A self-administered ISTH-BAT (self-BAT) would enhance screening and save time during an outpatient clinic visit.

**Aim** This study was aimed to investigate the reliability and feasibility of the self-BAT.

**Methods** The electronic self-BAT was created from the ISTH-BAT and paper-version of self-BAT and optimized by patients and physicians. Patients with a (suspected) congenital platelet defect (CPD), who had previously undergone physician-administered ISTH-BAT assessment, were invited to complete the self-BAT. Optimal self-BAT cut-off values to detect a bleeding tendency, as defined by the ISTH-BAT, were evaluated by receiver operator characteristic (ROC) curve analysis to reach a sensitivity ≥95%. Reliability was tested by assessing sensitivity, specificity, and intraclass correlation (ICC). Feasibility was evaluated on comprehension and length of self-BAT.

**Results** Both versions of the BAT were completed by 156 patients. Optimal cut-off values for self-BAT to define a bleeding tendency were found to be identical to those of the ISTH-BAT. Normal/abnormal scores of the ISTH-BAT and self-BAT were agreed in 88.5% (138/156, 95% confidence interval [CI]: 0.83–0.93) of patients. The sensitivity and specificity of the self-BAT to detect a bleeding tendency were 96.9 and 48.1%, respectively. The ICC was 0.73. Self-BAT questions were graded by 96.8% (151/156) as “very easy,” “easy,” and “satisfactory” and questionnaire length as “exactly right” by 91% (142/156) of patients.

**Conclusion** In patients with a (suspected) CPD, the self-BAT is sufficiently reliable and feasible to detect a bleeding tendency, which supports its use as a screening tool.

## Introduction

Bleeding disorders include von Willebrand disease (VWD), platelet disorders, hemophilia, and other clotting factor deficiencies. 1 Clinical symptoms range from continued bleeding after injury to severe spontaneous bleeding. Bleeding disorders can be difficult to diagnose due to inconclusive and expensive laboratory tests. Over the past few years, several standardized bleeding assessment tools (BATs) have been created to aid in distinguishing normal from abnormal bleeding during the diagnostic workup of a suspected bleeding disorder and to grade the bleeding severity. 2

In 2010, the International Society for Thrombosis and Hemostasis (ISTH) has endorsed a new BAT (ISTH-BAT), which is currently implemented in clinical practice widely to detect mild bleeding disorders and to assess the severity of the bleeding symptoms. 3 By completing this 14-domain questionnaire, a bleeding score is determined ranging from 0 to 56 points. The ISTH-BAT can be used for all types of congenital bleeding disorders and can be used to select patients in whom further laboratory investigations are necessary. 4 5 The ISTH-BAT has a high sensitivity and negative predictive value (NPV) for bleeding disorders that makes it suitable as a screening tool. 2 6

The ISTH-BAT is designed as a physician-administered questionnaire, yet completing the questionnaire is time consuming and requires expertise. 2 Recently, a self-administered ISTH-BAT (self-BAT) was generated in Canada. 7 Their aim was to generate, optimize, and validate a self-BAT as a screening tool for patients referred for suspected VWD. In the preliminary analysis, reliability of the self-BAT was considered excellent with an intraclass correlation coefficient (ICC) of 0.87 compared with the ISTH-BAT. In the final analysis, sensitivity and specificity of the self-BAT outcomes were found comparable to the ISTH-BAT, using laboratory-defined diagnosis of VWD as a reference standard.

Patients suspected of a congenital platelet disorder (CPD) comprise a large proportion of the patients seen at the outpatient clinic of hemophilia treatment centers (HTC). A (Dutch) self-BAT for this population is not yet available. HTC physicians conduct the ISTH-BAT as part of the standard procedure for bleeding assessment in the diagnostic workup of this population. A self-BAT completed at home would save valuable time during an outpatient clinic visit and enhance screening before referral to the HTC for a suspected CPD. Therefore, our aim was to assess the reliability and feasibility of the Dutch online self-BAT in patients referred for assessment of CPDs.

## Materials and Methods

### Study Participants

This study included patients who participated in the cross-sectional “Thrombocytopathy in the Netherlands” (TiN) study between February 2016 and December 2017 ( Supplement 1 ). 8 The TiN study investigated 201 patients with a bleeding tendency who were suspected of or had been diagnosed with a CPD. Assessment included the ISTH-BAT score (physician-administered), platelet characteristics and function, and DNA analysis. To validate the self-BAT in this cohort, all TiN patients were reinvited to complete the self-BAT questionnaire. Only TiN participants who had previously declined participation in future studies and deceased participants were excluded ( *n* = 3 and *n* = 1, respectively). Nonrespondents were contacted via telephone and e-mail. The ethics board of the University Medical Center Utrecht (UMCU) confirmed that the Medical Research Involving Human Subjects Act does not apply (reference number: 18-329).

### ISTH-Bleeding Assessment Tool

The 14 ISTH-BAT domains cover epistaxis, cutaneous bleeding, minor cutaneous wounds, hematuria, gastrointestinal bleeding, oral cavity bleeding, tooth extraction, surgical bleeding/major trauma, menorrhagia, postpartum bleeding, muscle hematomas, hemarthrosis, central nervous system bleeding, and one final domain on other bleeding symptoms. Each domain scores from 0 (absence of bleeding symptoms) to 4 (symptoms requiring extensive medical intervention), and the overall bleeding score is determined by summing the scores for all domains. 3 An abnormal bleeding score has been determined for men >3 and for women >5. 9

### Self-Administered ISTH-Bleeding Assessment Tool

At the beginning of 2016, the Dutch ISTH-BAT had been incorporated into the electronic patient records of UMCU and a Dutch self-BAT designed for children was back and forward translated by another institute, during which the diagnostic accuracy of this preliminary version of the self-BAT was evaluated by receiver operator characteristic (ROC) curves, using laboratory assessment as per the reference standard. 10 This preliminary self-BAT formed the basis of the current electronic self-BAT. The self-BAT was then tested by one pediatric and five adult hemophilia treating physicians of the Van Creveldkliniek and by two male and two female members of the Dutch Hemophilia Patient Organization. In addition, seven naive patients suspected of a bleeding disorder completed the self-BAT prior to their first visit to the clinic. Unclear self-BAT questions were adjusted till easy understanding was achieved. Medical terms were replaced by common words (such as epistaxis to nosebleeds) and at various points examples were given (muscle hematoma was explained by a description of the clinical symptoms; Self-Bat Dutch version can be provided on request). Once no noteworthy alterations were required, the self-BAT was sent to the TiN patients who had approved participating in this side study via a secured link by e-mail during July and August 2018. Each patient completed the self-BAT at home, without assistance. A reminder was circulated after 1 month in case of missing or incomplete responses. Incomplete questionnaires were excluded after study closure.

### Statistical Analysis

ISTH-BAT questionnaires from the previous TIN study were compared with the current self-BAT questionnaires. Incomplete self-BAT questionnaires were excluded from the analyses. To assess optimal cut-off values for men and women for the self-BAT, ROC curves were constructed, and a sensitivity of at least 95% to detect bleeding tendency defined by the ISTH-BAT was sought to avoid false negative bleeding assessments which are undesirable for a screening instrument. We hypothesized that the self-BAT score would be higher than the ISTH-BAT score, possibly warranting a different cut-off value for the self-BAT to define a bleeding tendency in accordance to the ISHT-BAT definitions. Sensitivity, specificity, positive predictive value (PPV), and negative predictive value (NPV) were assessed using the optimal cut-off scores for the self-BAT. Also the absolute agreement was calculated; we hypothesized that in less than 10% of cases, the outcomes of the surveys (normal or abnormal bleeding) would be discrepant.

For intersurvey reliability, we considered an ICC of >0.75 and limits of agreement (LoA) ± 3 (i.e., 10% of the ISTH-BAT range) between the two survey types as good. 11 A Bland–Altman plot was constructed to visualize the LoA. A sensitivity analysis was performed to detect any relevant differences between the TiN patients who did and who did not agree to participate in this self-BAT study. The influence of age, sex, and time between self-BAT and ISTH-BAT assessment on the difference between self-BAT and ISTH-BAT scores were visualized with scatter plots and analyzed using univariable and multivariable linear regression analysis. For comparison of the medians, the Wilcoxon signed rank test was used, considering a *p* -value of <0.05 as significant.

Feasibility was assessed from the patients' perspective on the time necessary to complete the questionnaire and the difficulty of questions (3- and 5-point Likert's scales). In addition, patients were asked to provide feedback on improvement and positive aspects of the questionnaire (free text). Data were analyzed using IBM SPSS Statistics Version 25 (IBM Corp., Armonk, New York, United States).

## Results

During the inclusion period, 197 patients were contacted from the TiN study. Those patients not willing to complete the self-BAT (9%, 17/197) and those patients who did not respond to our invitation (8%, 16/197), failed to submit (5%, 9/197), or completed (1%, 1/197) the questionnaire were excluded. The final cohort consisted of 156 patients, 78% (156/201) from the original TiN study. Baseline characteristics are depicted in Table 1 . Of the original TiN study population, 19 patients had previously been diagnosed with a CPD, whereas 182 were suspected of having a CPD. The currently included patients were 76.9% female, had a median age of 42.5 years, median ISTH-BAT total score of 10 (interquartile range (IQR): 7–14) with a final CPD diagnosis in 48.4% of patients. In comparison, TiN patients who did not participate in the self-BAT were 88.9% (40/45, *p* = 0.005) female, with a median age of 31.0 years ( *p* = 0.000), but comparable median ISTH-BAT total score of 9 (IQR: 7–11, *p* = 0.132) and final CPD diagnosis in 42.2% (19/45, *p* = 0.272).

**Table 1:** Baseline characteristics and BAT scores of included patients ( *n* = 156)

Age (y)	Median (range)	42.5 (18–76)
**Sex**	Female, *n* (%)	120 (76.9)
** Classification a**	Congenital platelet defect, *n* (%) Isolated thrombocytopenia ADP pathway defect Glanzmann thrombasthenia TxA2 pathway defect Dense granule deficiency Complex abnormalities b Bernard–Soulier syndrome Possible congenital platelet defect Acquired platelet defect Von Willebrand disease Unexplained bleeding tendency	77 (48.4) 20 (12.8) 15 (9.6) 13 (8.3) 11 (7.1) 8 (5.1) 6 (3.8) 4 (2.6) 16 (10.3) 3 (1.9) 1 (0.6) 59 (37.8)
**ISTH-BAT score**	Median (range) Score 0 (floor score), *n* (%) Score > 5 for women, *n* (%) Score > 3 for men, *n* (%)	10 (0–26) 5 (3.2) 104 (86.7) 25 (69.4)
**Self-BAT score**	Median (range) Score 0 (floor score), *n* (%) Score > 5 for women, *n* (%) Score > 3 for men, *n* (%)	12 (0–33) 2 (1.3) 110 (91.7) 29 (80.6)

Abbreviations: ADP, adenosine diphosphate; ISTH-BAT, International Society for Thrombosis and Hemostasis–bleeding assessment tool; self-BAT, self-administered ISTH-BAT; TxA2, Thromboxane A2.

a Final diagnosis at the end of the “Thrombocytopathy in the Netherlands” study.

b The patterns of platelet function defects did not support the diagnosis of one particular congenital platelet defect.

### Cut-off Values for the Self-BAT

**Fig. 1:**
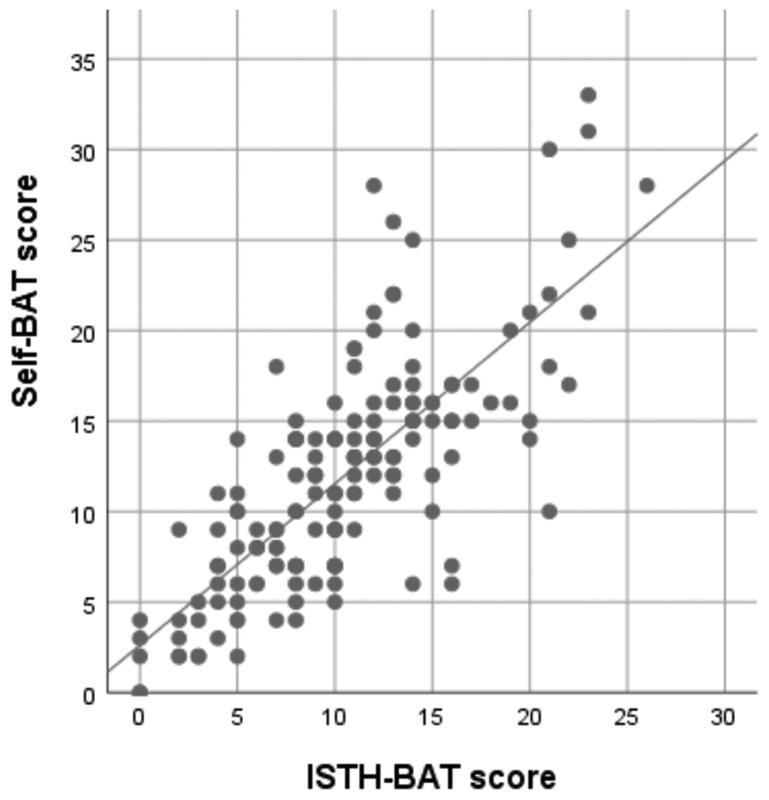
Scatter plot of self-BAT and ISTH-BAT scores. Y-axis: self-BAT score; X-axis: ISTH-BAT score; Linear model: self-BAT score = 2.62 + 0.89*(ISTH-BAT score). ISTH-BAT, International Society for Thrombosis and Hemostasis–bleeding assessment tool; self-BAT, self-administered ISTH-BAT.

The optimal cut-off values for the self-BAT were determined to be >3 for men and >5 for women, the same as for the original ISTH-BAT ( Supplement 2 ). 9 The self-BAT score was significantly higher than the ISTH-BAT score (median difference = 2.00, range = 16, Table 1 ; Fig. 1 ).

### Reliability

The sensitivity, specificity, PPV, and NPV of the self-BAT to detect a bleeding tendency were 96.9, 48.2, 89.9, and 76.5%, respectively ( Table 2 ). The normal/abnormal outcome classification of the ISTH-BAT and self-BAT were agreed in 138 of 156 (88.5%, 95% CI: 0.83–0.93) patients, slightly lower than our hypothesized agreement of 90%. The exact agreement percentages and agreement ±1 point per domain are shown in Table 3 and Supplement 3 . Both over- and underreporting of symptoms in the self-BAT compared with the ISTH-BAT was seen on the individual domains.

**Table 2:** Test performance of the Self-BAT

	Sensitivity in % (95% CI)	Specificity in % (95% CI)	PPV in % (95% CI)	NPV in % (95% CI)
Overall	96.9 (92.3–99.2)	48.2 (28.7–68.1)	89.9 (86.1–92.8)	76.5 (53.4–90.2)
Women	97.1 (91.8–99.4)	43.8 (19.8–70.1)	91.8 (87.9–94.5)	70.0 (40.1–89.0)
Men	96.0 (79.7–99.9)	54.6 (23.4–83.3)	82.8 (71.4–90.2)	85.7 (44.9–97.8)

Abbreviations: CI, confidence interval; NPV, negative predictive value; PPV, positive predictive value; self-BAT, self-administered ISTH-BAT.

Note: Cut-off value used was >5 for women and >3 for men.

**Table 3:** Percentage agreement between self-BAT and ISTH-BAT (domain) scores ( *n* = 156)

	ISTH-BAT	Self-BAT	% exact agreement a	% agreement ±1 b
**Outcome classification**				
Normal bleeding	27	17	NA	NA
Abnormal bleeding	129	139	NA	NA
**Score per domain, median (range)**				
1 Epistaxis	0 (0–4)	1 (0–4)	62.8	84.0
2 Cutaneous bleeding	0 (0–3)	0 (0–4)	57.7 d	82.7
3 Minor cutaneous wound	1 (0–3)	1 (0–4)	57.1 d	85.9
4 Hematuria	0 (0–4)	0 (0–3)	89.7	94.2
5 Gastrointestinal bleeding	0 (0–4)	0 (0–4)	87.8	91.7
6 Oral cavity bleeding	0 (0–4)	0 (0–4)	74.4	87.2
7 Tooth extraction	0 (0–4)	2 (0–4)	55.1 d	71.8 d
8 Surgical bleeding/major trauma	2 (0–4)	2 (0–4)	60.3	76.3 d
9 Menorrhagia	3 (0–4)	3 (0–4)	74.4	92.9
10 Postpartum bleeding	0 (0–4)	0 (0–4)	75.0	90.4
11 Muscle hematomas	0 (0–4)	0 (0–4)	86.5	93.6
12 Hemarthrosis	0 (0–3)	0 (0–4)	83.3	89.7
13 Central nervous system bleeding	0 (0–4)	0 (0–4)	98.1	98.7
14 Other bleeding symptoms c	0 (0–3)	1 (0–4)	54.5 d	89.7

Abbreviation: NA, not applicable.

a Percentage agreement is defined as exact same score.

b Percentage agreement is defined as same score or one point difference.

c Excessive umbilical stump bleeding, cephalohematoma, suction bleeding, venipuncture bleeding and bleeding during intercourse.

d Exact agreement percentage under the 60%, agreement ±1 under 80%.

**Fig. 2:**
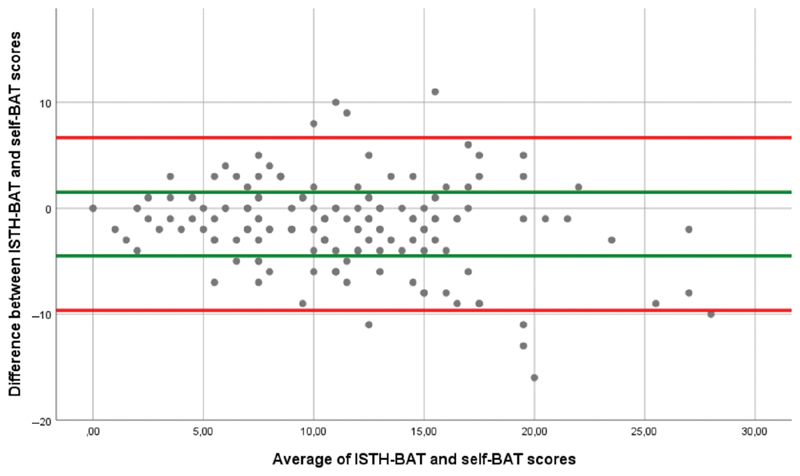
Bland–Altman plot of the differences between ISTH-BAT and self-BAT score versus the average of the two scores. Y-axis: difference in scores between self-BAT and ISTH-BAT; X-axis: average of the ISTH-BAT and self-BAT total score; limits of agreement (LoA) from study = red line, green line = ±3 from the average score which is the beforehand determined LoA: −9.65 to 6.67. ISTH-BAT, International Society for Thrombosis and Hemostasis–bleeding assessment tool; self-BAT, self-administered ISTH-BAT.

The Bland–Altman plot of the differences between ISTH-BAT and self-BAT score versus the average of the two scores is presented in Fig. 2 , with a LoA of −9.65 to 6.67, compared with our hypothesis of ±3. The ICC was 0.73 (95% CI: 0.64–0.79).

With increasing age, the self-BAT score showed an on average 0.059 (range: 0.029–0.088) per year increase based on the regression coefficient of multivariable linear regression analysis. Females had a somewhat larger absolute difference in scores (−0.001 [range: −1.092 to 1.091]). The time elapsed between both BAT assessments did not affect the difference between both scores (−0.001 [range: −0.001 to 0.003]; Supplement 4 ).

### Feasibility

The questions were graded as “very easy,” “easy,” and “satisfactory” in 96.8% (151 of 156) of the patients ( Table 4 ). The length of the questionnaire was experienced as “exactly right” in 91% (142 of 156) of the patients ( Table 5 ). Recurrent themes on suggestions for improvement where to add an “I don't know/I don't remember” option and free text to allow for comments and elaboration. Patients felt that free text would aid in cases where standard answers would not seem applicable, for example, when prophylaxes had been administered before surgery. Patients at an older age reported not being able to remember details of their bleeding history and women during/after menopause reported having difficulty in reporting their earlier menstrual cycle. Positive remarks focused on the clear and easy to understand nature of the questions.

**Table 4:** Evaluation of the difficulty of questions of the self-BAT

	Number of patients	% of patients
Very difficult	3	1.9
Difficult	2	1.3
Satisfactory	46	29.5
Easy	57	36.5
Very easy	48	30.8
**Total**	156	100.0

Abbreviation: self-BAT, self-administered ISTH-BAT.

**Table 5:** Evaluation of the length of the self-BAT

	Number of patients	% of patients
Too short	8	5.1
Exactly right	142	91.0
Too long	6	3.8
Total	156	100.0

Abbreviation: self-BAT, self-administered ISTH-BAT.

## Discussion

The present study assessed the reliability and feasibility of a self-BAT for screening of patients referred to a hemophilia treatment center with a suspected or known CPD. The optimal cut-off values for defining a bleeding tendency were found to be similar to those of the original ISTH-BAT. The self-BAT appears reliable with a sensitivity of 96.9% to detect a bleeding tendency. Patients valued the clear and easy to understand nature of the questions and the length of the questionnaire was experienced as appropriate.

This is the first study to evaluate reliability and feasibility of the self-BAT in patients referred for assessment of CPDs. A previous Canadian study evaluated the self-BAT in patients with VWD and showed that their self-BAT was a reliable screenings tool with an ICC of 0.87 and sensitivity of 78% to detect VWD. 7 The major difference between the Canadian self-BAT and the current self-BAT is the language (English vs. Dutch), the wording used and hard copy versus digital questionnaires, whereas the scoring system is similar. We found a higher sensitivity; however, this refers to the ability in detecting a bleeding tendency comparable to the ISTH-BAT, instead of detecting a particular bleeding disorder. Their cut-off values for the self-BAT to define a bleeding tendency were comparable to our study. The high sensitivity of 97% indicates very few false negative bleeding assessments in a population with a suspected or diagnosed CPD, a desirable feature of screening instruments. Even though the ISTH-BAT and self-BAT scores are highly correlated, a discrepancy of 11.5% of normal/abnormal cases and larger than expected LoA were found. The discrepant cases are bordering the cut-off value for a bleeding tendency, in very few cases, leading to a “false negative” score (2.5%, 4 of 156). The LoA discrepancy can be explained by the larger dispersion seen in higher self-BAT scores, far above the cut-off value for a bleeding tendency and thus of no implication for the diagnostic workup. On the contrary, a potential concern of the moderate specificity of 48% might be overdiagnosis and medicalization. The specificity could be increased if higher cut-off values were chosen, yet this would result in a loss of sensitivity (and thus the effectiveness as a screening tool for further diagnostic/laboratory workup). Lastly, relatively low NPV of the self-BAT in the current study is explained by the high prevalence of bleeding tendency in the investigated population. More research is needed to define evidence based criteria to safely exclude an inherited bleeding disorder with an ISTH-BAT score below the cut-off for an increased bleeding tendency. 2 6

Strengths of this study include the large cohort in which this self-BAT was evaluated and the extensive pretesting in health care workers and naïve patients. A limitation of this study is the overrepresentation of female patients and the lack of an accurate gold-standard test. The participation bias (older patients and more females) could have influenced the results, although the median ISTH-BAT scores and proportion of patient with a bleeding tendency did not differ in the TiN patients who declined participating of this self-BAT study. We compared the data of our self-BAT to the ISTH-BAT, although the ISTH-BAT is recommended for clinical use, the test–retest reliability of both BATs has not yet been evaluated.

We recommend the use of the self-BAT in the clinic, but results should be discussed and interpreted with caution during consultation. Higher self-administered scores can occur when patients interpret normal bleeding events as abnormal. Discrepant cases (normal/abnormal bleeding) commonly differed by only a few points on the total score, but resulted in >10% of the cases in a discrepant outcome. The ICC of 0.73 is acceptable and can be explained by the dispersion seen in higher scores. Although a larger dispersion in differences between BATs was seen in the elderly, the dispersion was symmetric, and therefore it is unlikely that recall bias caused the discrepancies. By discussing the self-BAT results with a physician during the following outpatient clinic visit, scores can be adjusted accordingly. This would still be more time efficient than the current ISTH-BAT and coincides with its use as a screening tool and preliminary assessment of bleeding history.

Future studies on secondary and primary care centers are needed to assess potential use of the self-BAT in these settings and to assess the potential for its use as a screening instrument for clinically relevant bleeding disorders in general. In conclusion, the self-BAT has a high sensitivity to detect a bleeding tendency as defined by the current standard ISTH-BAT, which supports its use as a screening tool, and was valued by patients for its clear questions and appropriate length.
