# Supplementary material for: Reliability and Feasibility of the Self-Administered ISTH-Bleeding Assessment Tool
Source: TH Open. 2019 Nov 29;3(4):e350–5. doi: 10.1055/s-0039-3400483 (PMC6884422; doi:10.1055/s-0039-3400483)

Supplementary material

Supplement 1. Inclusion and exclusion criteria for “Thrombocytopathy in the Netherlands” (TiN) study.

The following patients were eligible to participate in the TiN study:

1. Patients with a previously identified congenital platelet defect (CPD).
2. Patients suspected for a CPD based on previous abnormal platelet counts, light transmission aggregometry (LTA) results, or platelet ADP content.
3. Patients suspected for a CPD based on a predominantly mucocutaneous bleeding tendency compatible with a CPD, in whom other known causes of bleeding were excluded, and in whom previous LTA results or platelet ADP content were normal.
4. Patients suspected for a CPD based on a predominantly mucocutaneous bleeding tendency compatible with a CPD, in whom other known causes of bleeding were excluded, newly referred for platelet function testing.

Exclusion criteria were use of antiplatelet drugs that could not be stopped prior to inclusion, a known acquired platelet disorder, von Willebrand disease, hemophilia or another disorder of secondary hemostasis or fibrinolysis, and inability to give informed consent.

Supplement 2: Receiver operator characteristic (ROC) curves International Society for Thrombosis and Hemostasis-bleeding assessment tool (ISTH-BAT) and self-administered ISTH-BAT (self-BAT).

Coordinates of ROC curve for men			
Positive if ≥ to	Sensitivity	1-Specificity	Specificity
−1.00	1.000	1.000	0
1.00	1.000	0.818	0.182
2.50	1.000	0.545	0.455
3.50	0.960	0.455	0.545
4.50	0.920	0.182	0.818
5.50	0.880	0.091	0.909
6.50	0.680	0.091	0.909
8.00	0.560	0.091	0.909
9.50	0.560	0.000	1
11.00	0.480	0.000	1
13.00	0.440	0.000	1
14.50	0.360	0.000	1
15.50	0.240	0.000	1
16.50	0.200	0.000	1
17.50	0.160	0.000	1
19.50	0.120	0.000	1
21.50	0.080	0.000	1
24.00	0.040	0.000	1
27.00	0.000	0.000	1

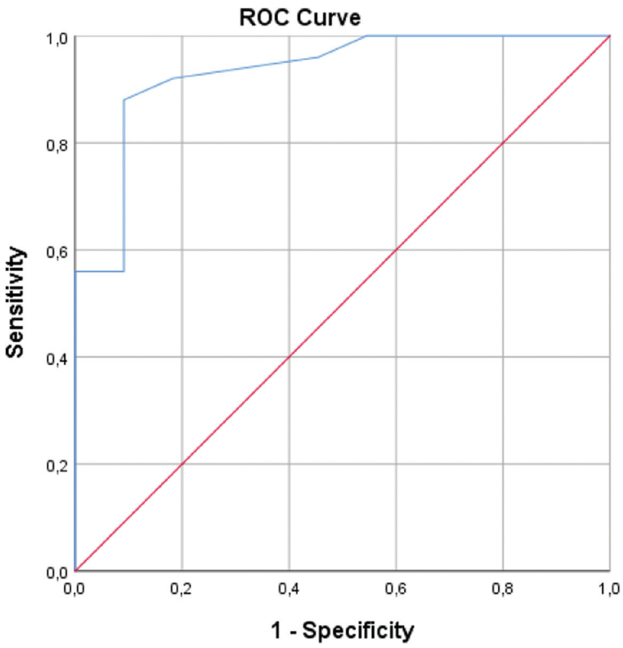

Coordinates of ROC curve for women			
Positive if ≥ to	Sensitivity	1-Specificity	Specificity
1.00	1.000	1.000	0
2.50	1.000	0.813	0.188
3.50	1.000	0.750	0.250
4.50	0.981	0.688	0.313
5.50	0.971	0.563	0.438
6.50	0.942	0.500	0.500
7.50	0.875	0.375	0.625
8.50	0.837	0.313	0.688
9.50	0.760	0.250	0.750
10.50	0.731	0.125	0.875
11.50	0.663	0.000	1
12.50	0.587	0.000	1
13.50	0.490	0.000	1
14.50	0.385	0.000	1
15.50	0.308	0.000	1
16.50	0.231	0.000	1
17.50	0.183	0.000	1
18.50	0.154	0.000	1
19.50	0.135	0.000	1
20.50	0.106	0.000	1
21.50	0.087	0.000	1
23.50	0.067	0.000	1
26.50	0.048	0.000	1
29.00	0.029	0.000	1
30.50	0.019	0.000	1
32.00	0.010	0.000	1
34.00	0.000	0.000	1

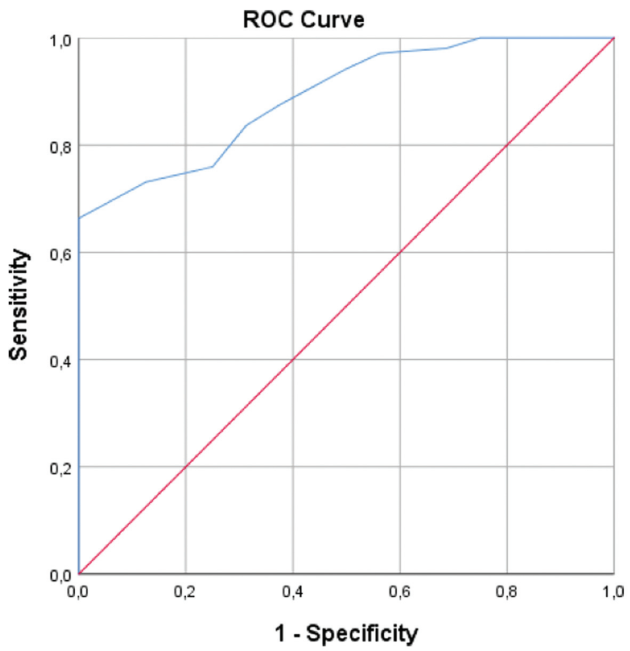

Supplement 3. Scatter plot and crosstable of International Society for Thrombosis and Hemostasis-bleeding assessment tool (ISTH-BAT) domain score versus self-administered ISTH-BAT (self-BAT) domain score.

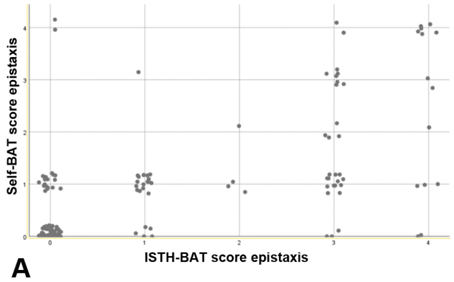

A

Epistaxis

		Self-BAT score					
		0	1	2	3	4	Total
ISTH-BAT score	0	68	17	0	0	2	87
	1	5	16	0	1	0	22
	2	0	3	1	0	0	4
	3	3	13	4	7	2	29
	4	2	3	1	2	6	14
Total		78	52	6	10	10	156

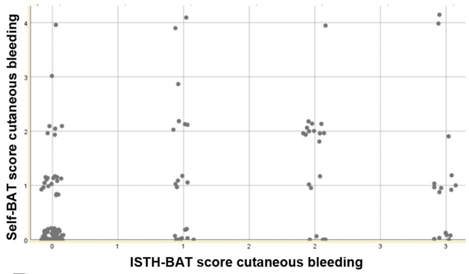

B

Cutaneous bleeding

		Self-BAT score					
		0	1	2	3	4	Total
ISTH-BAT score	0	74	16	5	1	1	97
	1	11	5	4	1	2	23
	2	5	3	11	0	1	20
	3	6	7	1	0	2	16
	Total	96	31	21	2	6	156

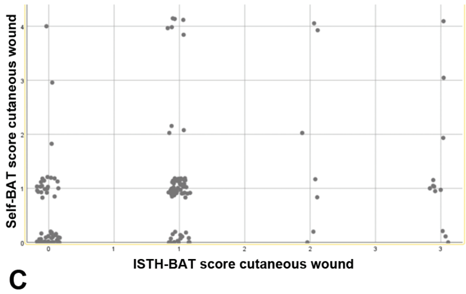

C

Cutaneous wound

		Self-BAT score					
		0	1	2	3	4	Total
ISTH-BAT score	0	38	20	1	1	1	61
	1	18	49	3	0	6	76
	2	2	2	1	0	2	7
	3	3	6	1	1	1	12
	Total	61	77	6	2	10	156

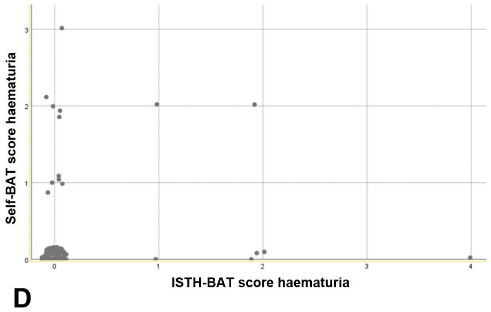

D

Hematuria

		Self-BAT score 0	1	2	3	Total
ISTH-BAT score	0	139	5	4	1	149
	1	1	0	1	0	2
	2	3	0	1	0	4
	4	1	0	0	0	1
Total		144	5	6	1	156

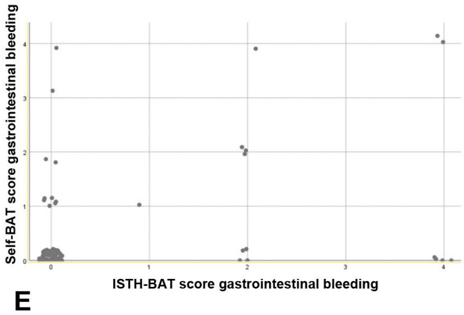

E

Gastrointestinal bleeding

		Self-BAT score 0	1	2	3	4	Total
ISTH-BAT score	0	131	6	2	1	1	141
	1	0	1	0	0	0	1
	2	4	0	3	0	1	8
	4	4	0	0	0	2	6
Total		139	7	5	1	4	156

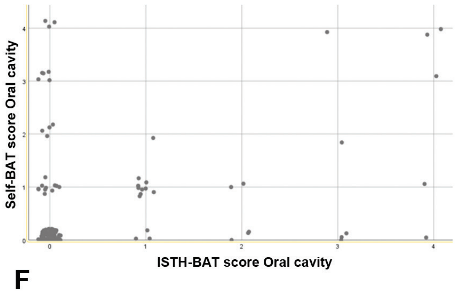

F

Oral cavity

		Self-BAT score 0	1	2	3	4	Total
ISTH-BAT score	0	105	11	4	5	3	128
	1	3	9	1	0	0	13
	2	3	2	0	0	0	5
	3	3	0	1	0	1	5
	4	1	1	0	1	2	5
Total		115	23	6	6	6	156

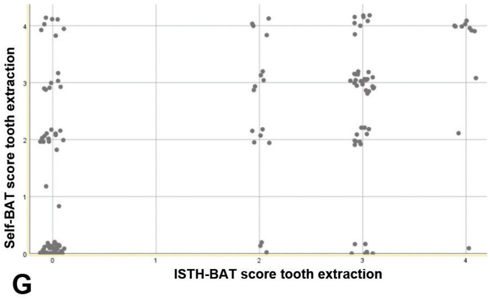

G

Tooth extraction

		Self-BAT score					
		0	1	2	3	4	Total
ISTH-BAT score	0	49	2	14	7	7	79
	2	3	0	5	5	4	17
	3	7	0	10	24	8	49
	4	1	0	1	1	8	11
	Total	60	2	30	37	27	156

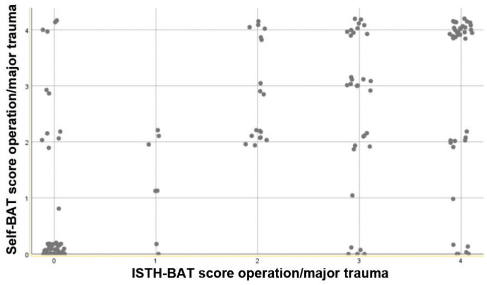

H

Operation/major trauma

		Self-BAT score					
		0	1	2	3	4	Total
ISTH-BAT score	0	47	1	5	2	4	59
	1	2	2	3	0	0	7
	2	0	0	9	3	6	18
	3	5	1	6	9	10	31
	4	6	1	7	0	27	41
	Total	60	5	30	14	47	156

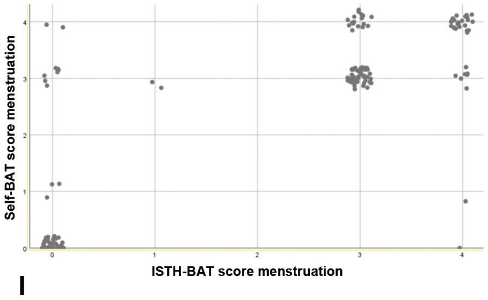

I

Menstruation

		Self-BAT score				
		0	1	3	4	Total
ISTH-BAT score	0	43	3	7	2	55
	1	0	0	2	0	2
	3	0	0	48	17	65
	4	1	1	7	25	34
	Total	44	4	64	44	156

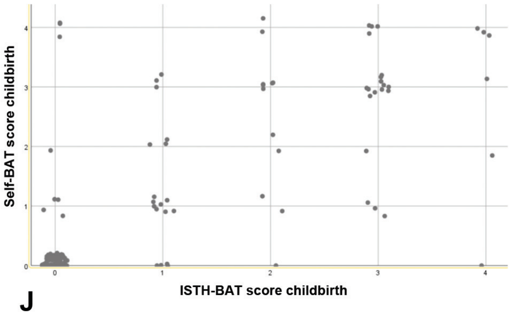

J

Childbirth

		Self-BAT score					
		0	1	2	3	4	Total
ISTH-BAT score	0	93	4	1	0	3	101
	1	4	8	3	3	0	18
	2	1	2	2	5	2	12
	3	0	3	1	11	4	19
	4	1	0	1	1	3	6
	Total	99	17	8	20	12	156

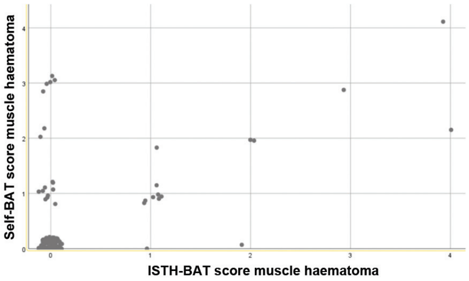

K

Muscle hematoma

		Self-BAT score					
		0	1	2	3	4	Total
ISTH-BAT score	0	124	10	2	5	0	141
	1	1	7	1	0	0	9
	2	1	0	2	0	0	3
	3	0	0	0	1	0	1
	4	0	0	1	0	1	2
	Total	126	17	6	6	1	156

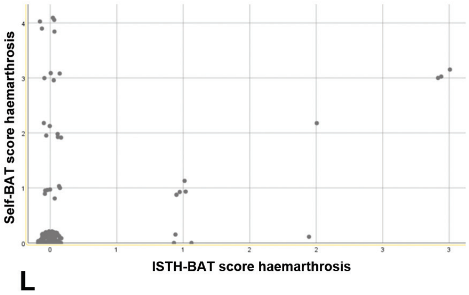

L

Haemarthrosis

		Self-BAT score					Total
		0	1	2	3	4	
ISTH-BAT score	0	122	7	6	4	5	144
	1	3	4	0	0	0	7
	2	1	0	1	0	0	2
	3	0	0	0	3	0	3
	Total	126	11	7	7	5	156

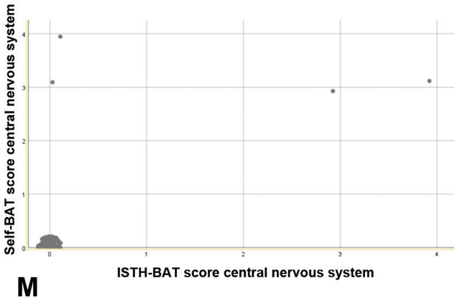

M

Central nervous system

		Self-BAT score			
		0	3	4	Total
ISTH-BAT score	0	152	1	1	154
	3	0	1	0	1
	4	0	1	0	1
	Total	152	3	1	156

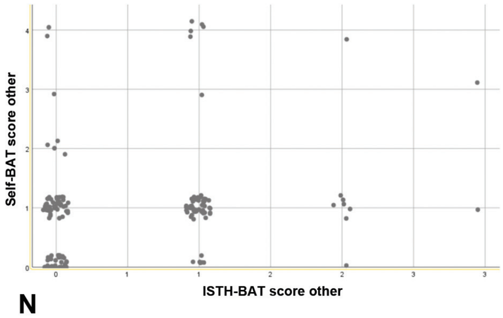

N

Other

		Self-BAT score					
		0	1	2	3	4	Total
ISTH-BAT score	0	40	43	4	1	2	90
	1	6	44	0	1	5	56
	2	1	6	0	0	1	8
	3	0	1	0	1	0	2
	Total	47	94	4	3	8	156

Supplement 4. (A) Effect of age, (B) sex, and (C) time between self-administered International Society for Thrombosis and Hemostasis (ISTH)-bleeding assessment tool (self-BAT) and ISTH-BAT assessment on the difference between self-BAT and ISTH-BAT score.

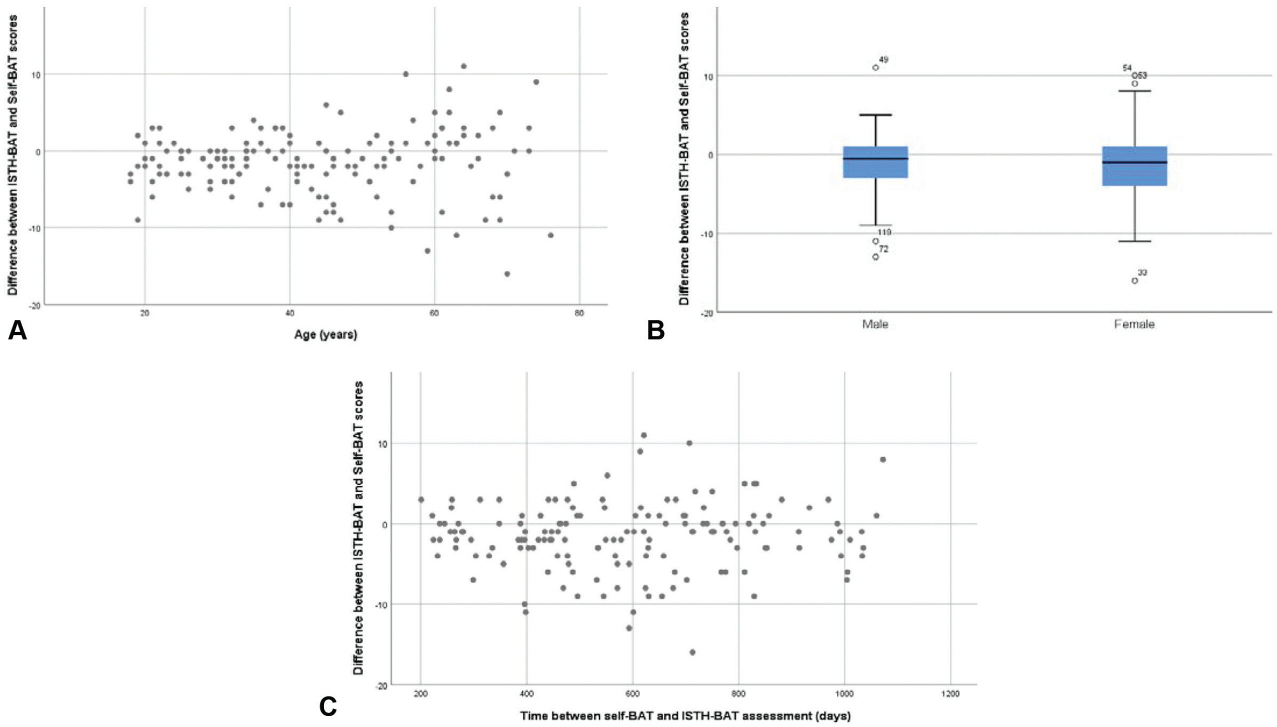

Supplement: Supplementary file 1 — Supplementary Material [file 10-1055-s-0039-3400483-s190050oa.pdf]
